# The Association between Perceived Annoyances in the Indoor Home Environment and Respiratory Infections: A Danish Cohort Study with up to 19 Years of Follow-Up

**DOI:** 10.3390/ijerph20031911

**Published:** 2023-01-20

**Authors:** Anne Marie Kirkegaard, Stine Kloster, Michael Davidsen, Anne Illemann Christensen, Jørgen Vestbo, Niss Skov Nielsen, Annette Kjær Ersbøll, Lars Gunnarsen

**Affiliations:** 1 Department of the Built Environment, Aalborg University, A.C. Meyers Vaenge 15, 2450 Copenhagen, Denmark; 2 National Institute of Public Health, University of Southern Denmark, Studiestraede 6, 1455 Copenhagen, Denmark; 3 Division of Infection, Immunity and Respiratory Medicine, University of Manchester, Manchester M13 9 PL, UK

**Keywords:** perceived annoyances, indoor environment, respiratory infection, environmental epidemiology

## Abstract

The increasing prevalence of reported annoyances in the indoor environment threatens public health. This study aimed to investigate the association between perceived annoyances from the home environment and respiratory infections among individuals with and without asthma or chronic obstructive pulmonary disease (COPD). A total of 16,688 individuals from the Danish Health and Morbidity Survey initiated in 2000 were grouped according to their patterns of perceived annoyances. Information on respiratory infections (all causes, bacterial, viral, and those leading to hospital admissions) was obtained from Danish registers up to 19 years after the survey. Poisson regression of incidence rates (IRs) was applied to estimate incidence rate ratios (IRRs). Annoyances significantly increased the IR for respiratory infections of all causes and bacterial respiratory infections in individuals without asthma or COPD, adjusted IRR 1.16 (95% CI: 1.01, 1.34) and 1.15 (95% CI: 1.02, 1.31), respectively. However, no difference was observed for viral respiratory infections nor hospital admissions. Individuals with asthma or COPD and a high level of annoyances had a non-significantly increased IR in all four analyses of respiratory infections. These findings provide support for perceived annoyances as an important risk factor for respiratory infections.

## 1. Introduction

The prevalence of reported indoor environmental annoyances at home has increased steadily over the past two decades [1,2,3,4,5,6]. Sensory annoyances and discomfort may occur as a result of poor indoor air, lighting, acoustics, or thermal conditions [7]. Environmental annoyances have been linked to adverse health effects, such as poor mental health, elevated blood pressure, and cardiovascular disease [8,9,10,11]. However, further evidence of links to respiratory infections is needed. Globally, respiratory infections are the most common illness [12] and respiratory infections acquired indoors account for almost all cases [13].

Exposure to indoor contaminants may affect the risk of respiratory infections, either directly [13,14] or indirectly, e.g., via annoyances [15,16,17,18]. Annoyances are emotional responses based on perceptions [19] that may initiate stress reactions, which, if experienced chronically, may trigger the onset of a variety of diseases [8,9,18,20,21].

Respiratory infections acquired indoors are caused primary by viruses [22] and secondarily by bacteria [22,23]. The three main sources of viruses and bacteria in the indoor environment are humans, microbial growth from water sources, and those brought indoors from the outdoor environment [24,25]. The balance between microbial environment, host, and environmental triggers influences the susceptibility to respiratory infections, phenotype, and severity [26]. Environmental triggers include viral infections, while non-infectious triggers include smoke, air pollution, and allergens [26]. Viral infections are most often mild and self-limited illnesses [27], thus rarely diagnosed. The risk of bacterial infections increases due to viral damage of the respiratory tract [28]. Individuals with asthma or chronic obstructive pulmonary disease (COPD) have significantly increased risk of common and serious viral and bacterial respiratory infections compared to individuals without asthma or COPD [29,30,31]. To our knowledge, no studies published to date have accounted for asthma or COPD status. Thus, it is unclear to what degree the association exists when differentiating based on asthma/COPD status, e.g., poorer airway defences among individuals with asthma and COPD could explain the observed association. Therefore, previous studies suffer from a problem of generalisability.

The few studies that have investigated the impact of indoor perceptions and annoyances on respiratory infections employed exposures as annoyances, such as temperature [16,32], draught [16], humidity [32], indoor air quality [15,18], pollution from traffic or industry [15], and noise (traffic and neighbourhood) [17]. Thus, the link between perceived indoor annoyances and respiratory infections has yet to be conclusively established.

The purpose of this study was to investigate the association between perceived annoyances from the indoor environment and (i) respiratory infections of all causes, (ii) bacterial infections, (iii) viral infections, and (iv) hospital admissions caused by respiratory infections among individuals with and without asthma or COPD, respectively.

## 2. Materials and Methods

### 2.1. Study Design

We designed a cohort study of participants from the Danish Health and Morbidity Survey during the period from enrolment in 2000 to the end of study on 31 December 2018. At enrolment, participants completed an interviewer-assisted survey in their home with information on health and morbidity [33], as well as factors with a potential impact on health status [34,35]. The 2000 survey had a special focus on the home environment and included detailed information on perceived annoyances [36]. Information was additionally obtained from Danish national registers to characterise participants and their dwellings from baseline until the end of follow-up.

### 2.2. Study Population

A total of 16,688 individuals participated in the Danish Health and Morbidity Survey in 2000 [33,34]. In brief, the eligibility criteria included individuals aged ≥16 years because adolescents aged ≥16 years were considered to have a similar understanding of the questions as adults. Moreover, individuals should have a residential address in Denmark [33]. The invited individuals were a representative sample of the population. Participants were enrolled in three rounds during 2000, in winter, spring, and autumn [33]. The design has been described elsewhere (see Davidsen and Kjøller, 2002 [33]).

### 2.3. Assessment of Perceived Annoyances of Indoor Environment

We determined the levels of perceived annoyances in the indoor environment at home based on 13 items about the perceived indoor environment reported by participants in the Danish Health and Morbidity Survey, including twelve items among the perceived annoyances (yes annoyed/no) within the last two weeks that covered indoor air, lighting, acoustics, and thermal comfort, and the item ‘Do you live next to a trafficked road?’ (yes/no). Items concerned with the indoor thermal, air quality, noise, and light environment were chosen as reported by the World Health Organization describing these four areas of great importance for the health of people indoors [37].

Perceived annoyances were specifically modelled using Latent Class Analysis. Based on this method, the study population was divided into three groups of individuals with similarities in their indoor environment, corresponding to low, moderate, and high levels of perceived annoyances. The material and methods have been described in detail elsewhere (see Kloster et al., 2022 [38]).

### 2.4. Respiratory Infection Outcomes

Information about respiratory infections was obtained from two Danish national registers. First, the Danish National Patient Registry contains individual-level data on inpatient, outpatient, and emergency department contacts in Denmark since 1977 [39]. Second, the Danish National Prescription Registry contains information on all redeemed antibiotic prescriptions filled at community pharmacies in Denmark since 1995 [40].

Respiratory infections of all causes were based on the International Classification of Diseases and Related Health Problems, 10th revision (ICD-10) codes and the Anatomical Therapeutic Chemical (ATC) classification codes. ICD-10 codes for respiratory infections were included independently of being the primary or secondary cause of hospital contact.

We identified respiratory infections by hospital contact (ICD-10 codes: J00–06, J09–18, J20–22, R05, and R07.0) and prescription (ATC codes: R01*, R02*, R03*, R05*, R06*, R07* (*including all subcategories), and J01CE02) (Table S1). The prescriptions had few exceptions for which they did not count as respiratory infection events based on the individual’s previous disease historic. For example, R01 did not count as a respiratory infection event if the individual had allergic rhinitis, asthma/COPD, or had more than one prescription within the last year (see exceptions in Table S1).

More than one diagnosis and/or drug prescription redemption for respiratory infections within 28 days for the same individual were regarded as the same event and therefore only counted once, using the initial date as the event date. However, after the occurrence of a respiratory infection event, an individual would be categorised as out of risk the following 28 days. For example, if an individual had an event on day 1 and another on day 12, this would be regarded as one event and the individual would be at risk again on day 41.

Four respiratory infection outcomes were investigated: all, bacterial, viral, and those causing hospital admissions. Bacterial respiratory infections were defined as redeemed antibacterial prescription drugs within the first 10 days after an initial event regardless of hospital contact (i.e., ATC code). Viral respiratory infections were defined by diagnosis at hospitals without any prescription drugs (i.e., ICD code and no ATC code) within the first 10 days after an initial event. We used 10 days as the cut-off point to limit the risk of individuals with viral respiratory infections getting co-bacterial infections and vice versa. Hospital admissions were defined by part or full-day admission (i.e., non-ambulant or emergency contact).

### 2.5. Confounders and Other Variables

We identified potential confounders such as age, sex, highest completed educational level, smoking status, ethnicity, number of residents, heating source, residential density, year of dwelling construction, type of dwelling, and degree of urbanisation. These were identified using a Directed Acyclic Graph (DAG) (Figure S1) created with DAG software [41]. Furthermore, we adjusted for the season the survey was completed, calendar season and year, number of years lived in the dwelling before the survey, and number of respiratory infections since study entry. In the analyses of the population with asthma or COPD, we further adjusted for baseline asthma/COPD diagnosis.

Information on age (<20, 20–24, 25–29, 30–34, 35–39, 40–44, 45–49, 50–54, 55–59, 60–64, 65–69, 70–74, 75–79, 80–84, ≥85 years old), sex (male or female), and ethnicity (emigrants and descendants or ethnic Danish) were obtained from Danish Civil Registration System [42]. Furthermore, the number of years lived in the dwelling before the survey (<3, 3–7, 8–12, 13–20, ≥21 years). Number of residents (1, 2, 3, 4, and ≥5) were obtained from the Population Statistics Register and we supplemented with data from the Danish Health and Morbidity Survey in case of missing data [4].

The highest completed educational level was updated yearly and obtained from the Danish Education Register [43], classified according to the International Standard Classification of Education System (ISCED) [44] and further aggregated into three groups: elementary (pre-primary, primary and lower secondary; ISCED level 1–2), short (upper secondary and postsecondary; ISCED level 3–4), and medium/long (tertiary education; ISCED level 5–8). If data were missing for one or more years, information was filled forward with data from the register, and if observations had missing data on the first or all years, they were enriched with information from the survey.

Dwelling type (detached house, semi-detached and terrace house, apartments, farmhouses, and others), smoking status (current or non-smoker), and the season the survey was completed (autumn/winter or spring/summer) were obtained from the Danish Health and Morbidity Survey [4].

Baseline information from Statistics Denmark was obtained for degree of urbanisation (town/city with <200, 200–4900, 5–49,000, ≥50,000 residents, missing), year of dwelling construction (<1960, 1960–1978, >1978, missing), and dwelling area. Dwelling area was used to calculate residential density (<40, 40–79, ≥80 m^2^) yearly by dividing the number of residents with the dwelling area.

The event date was used to create variables for the season (autumn/winter, spring/summer) and calendar year. Furthermore, we counted the number of previous events for each participant from baseline until end of risk.

Information about asthma and COPD was obtained from the Danish National Patient Registry and Danish National Prescription Registry until 31 December 2018 (Table S2). Information about cystic fibrosis (International Classification of Diseases, 8th revision (ICD-8): 273.09; ICD-10: E84) was obtained from the Danish National Patient Registry until 31 December 2018.

### 2.6. Risk Time

Individuals were ineligible if they had a diagnosis of cystic fibrosis and were excluded at baseline or censored at time of diagnosis, whichever came first.

The risk time for respiratory infections began on the date of interview. Participants who moved in within 28 days of the interview had their risk time started after 28 days. The 28-day period was to ensure that a potential respiratory infection could be attributed to the current environment. Risk time continued until participants moved, emigrated, died, were diagnosed with cystic fibrosis, or the end of follow-up on 31 December 2018, whichever came first. Individuals who were diagnosed with asthma or COPD during the study period, immediately changed to the group with asthma or COPD and the risk time was reset.

### 2.7. Statistical Analysis

We described the baseline characteristics for the study population in total and by level of perceived annoyances using frequencies and percentages for categorical variables. Mean follow-up time was calculated for individuals with and without asthma and COPD.

The incidence rate ratios (IRRs) for respiratory infections (all, bacterial, viral, and hospital admissions) were estimated in a generalised mixed model with the Poisson distribution of number of respiratory infections [45]. We compared highly and moderately annoyed individuals with those with the lowest level of annoyances. We applied a logarithmic transformation of risk time as offset. The risk time was split into distinct periods by calendar year, season, and age. The underlying assumption was that incidence rates of respiratory infections did not differ within each time period in the analysis [46]. A random (i.e., mixed) effect was applied to consider the correlation between recurrent respiratory infections in the same individual. To reduce the possible impact of non-response bias, we applied weights computed by Statistics Denmark based on information such as sex, age, education, and income [47,48].

Results were estimated in crude and adjusted models. None of the analyses were adjusted for ethnicity or heating source, as almost the entire population were Danes (98%) and heating source had 53% missing data, thus the validity of the latter was questionable.

Several sensitivity analyses were performed. First, to examine the effect of another outcome definition, we included four additional prescriptions to the existing outcome definition: the macrolides J01FA01, J01FA06, J01FA09, and J01FA10. Macrolides are mainly prescribed to individuals who are allergic to penicillin or for treatment of mycoplasma pneumonia [49,50,51]. Second, the effects of baseline asthma and COPD were evaluated by only including individuals with asthma or COPD at study entry. Third, we included only individuals who were diagnosed after enrolment.

Data management and descriptive analyses were performed in RStudio version 4.1.3 (Posit Software, PBC, Boston, MA, USA) and Poisson regressions were performed using STATA software version 17.0 (StataCorp LLC., College Station, TX, USA).

## 3. Results

### 3.1. Study Participants

**Figure 1:**
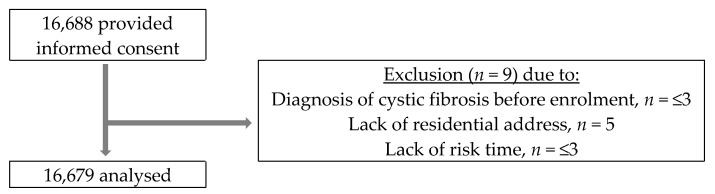
Flow diagram of cohort selection from the Danish Health and Morbidity Survey 2000.

A total of 16,679 individuals were included in the cohort and followed for a total of 155,438 person years. We excluded nine individuals because of a diagnosis of cystic fibrosis before enrolment (*n* = ≤3), no residential address at enrolment (*n* = 5), and no available risk time (*n* = ≤3), as shown in Figure 1.

A total of 15,521 individuals did not have a diagnosis of asthma or COPD at baseline, 1158 individuals had either asthma or COPD at baseline, and 1157 individuals were diagnosed with asthma or COPD during the study period.

Baseline characteristics differed between individuals at each level of perceived annoyances (Table 1). Individuals who perceived a high level of annoyances were more likely to be women, younger than 35 years old, current smokers, and had lived in their home for less than three years. Furthermore, individuals who were highly annoyed were less likely to live in a detached house, more likely to live in a city with more than 50,000 residents, have a residential density of ≥80 m^2^/resident, and live alone. A higher proportion of highly annoyed individuals was interviewed in winter than individuals with low and moderate levels of annoyances (Table 1).

### 3.2. Follow-Up Time and Number of Respiratory Infections

A total of 1619 and 15,408 respiratory infections of all causes occurred between baseline and censoring for the population with and without asthma or COPD, respectively, corresponding to an IR of 9.1 and 11.3 per 100 person years, respectively. The mean follow-up was 7.75 and 8.86 years for individuals with and without asthma or COPD, respectively.

### 3.3. Associations between Perceived Annoyances and Respiratory Infections

We found an association between perceived annoyances and respiratory infections of all causes among individuals without asthma and COPD, with an adjusted IRR of 1.16 (95% CI: 1.01, 1.34), when comparing the highest level of perceived annoyances with the lowest level (Table 2). The adjusted IRR for moderate level of annoyances was 1.15 (95% CI: 1.02, 1.31). The IRRs were almost similar for bacterial respiratory infections (Table 2). We observed no significant association with viral respiratory infections nor hospital admissions, where the adjusted IRRs for high and moderate levels of annoyances were decreased compared to that for the low level of annoyances (Table 2).

There were no significant associations between perceived annoyances and respiratory infections among individuals with asthma or COPD. The IRR for respiratory infections of all causes for individuals with asthma or COPD was increased for the high level of annoyances, with an adjusted IRR of 1.20 (95% CI: 0.85, 1.69), and decreased for the moderate level of annoyances, with an adjusted IRR of 0.84 (95% CI: 0.59, 1.21), compared to the low level of annoyances (Table 3). We observed almost the same estimates for the IRRs for bacterial respiratory infections (Table 3). A high level of annoyances increased the IRR for viral infections (adjusted IRR 1.28, 95% CI: 0.68, 2.42) and a moderate level of annoyances decreased the IRR (adjusted IRR 0.76, 95% CI: 0.38, 1.49), compared to the low level of annoyances (Table 3). For hospital admissions, with fewest cases, a high level of annoyances increased the adjusted IRR to 1.31 (95% CI: 0.68, 2.53) and a moderate level of annoyances decreased the adjusted IRR to 0.79 (95% CI: 0.39, 1.61), compared to the low level of annoyances (Table 3).

### 3.4. Sensitivity Analyses

Including four additional prescription drugs to the outcome for every analysis (in total six analyses; all events, bacterial events, and virus events for individuals with and without asthma or COPD, respectively) did not remarkably change the estimates. The sensitivity analyses had adjusted IRRs slightly closer to 1 than the main analyses.

The analyses of the asthma and COPD groups were repeated among individuals with diagnosis at enrolment and those who were diagnosed later. The results showed that individuals who were diagnosed after baseline with asthma or COPD and perceived a high level of annoyances had a higher adjusted IRR for respiratory infections (IRRs 1.56–1.87) than individuals with baseline asthma or COPD who were highly annoyed (IRRs 1.03–1.08), when compared against the respective groups with the low level of annoyances. However, the findings remained non-significant.

## 4. Discussion

### 4.1. Summary of Findings

In this cohort study with up to 19 years of follow-up, we found that individuals without asthma or COPD had significantly increased IRs for respiratory infections of all causes and bacterial respiratory infections for high and moderate levels of annoyances compared with a low level. In contrast, for viral infections and hospital admissions, individuals with high and moderate levels of annoyances had a non-significantly decreased IR. Individuals with asthma or COPD who perceived a high level of annoyances had non-significantly increased IRs for all four types of respiratory infections, while IRs of individuals with a moderate level of annoyances were non-significantly decreased compared to the IRs of those with a low level of annoyances.

### 4.2. Comparison with Other Studies

Few studies have investigated the association between single indoor environmental perceptions and respiratory infections among adults [15,16,17,18,32]. No previous research in this area has combined multiple annoyances in the indoor environment to investigate their association with respiratory infections. Thus, comparisons to other studies should be done with caution.

A recent study found that the perception of too high indoor temperatures during summer and experiencing draught in winter were significantly associated with having had at least one respiratory tract infection within the last year (odds ratio (OR) 1.54, 95% CI: 1.24, 1.91, and OR 1.76, 95% CI: 1.30, 2.39) [16]. These findings are in line with our results, as we observed that a higher level of perceived annoyances was significantly associated with more incidences of respiratory infections of all causes. In contrast, Quinn and Shaman found that a higher incidence of viral infection cases was neither associated with temperature perception (adjusted OR 0.73, *p* > 0.05), nor with humidity perception (adjusted OR 0.90, *p* > 0.05) [32]. Even though our finding for viral infections was consistent with that of Quinn and Shaman, our finding may be explained by the low incidence of viral infections in the study.

A previous study found that adults who were severely annoyed by general neighbourhood noise had significantly increased odds for bronchitis (OR 1.63) compared with adults who did not experience annoyances [17]. Furthermore, severe annoyance by general traffic noise (road, aircraft and railway noise) was significantly associated with bronchitis (OR 1.68) compared to no annoyance [17]. Disturbance by air pollution from traffic or industry when the windows were open was associated with respiratory infections (adjusted prevalence OR 1.76, 95% CI: 0.49, 6.34) [15]. In our study, noise from traffic and living next to a trafficked road were especially prevalent among individuals who perceived moderate and high levels of annoyances, which most likely correlated with air pollution [38]. The risk of respiratory infections have also been found to increase non-significantly with poor perceived indoor air quality (adjusted prevalence OR 2.47, 95% CI: 0.68, 8.94) [15]. Further, residents who reported odour annoyances had non-significantly increased odds for having had a respiratory infection during the preceding 12 months (fully adjusted OR 1.2, 95% CI: 0.9, 1.7) compared to residents with no annoyances [18]. In our study, annoyance by stuffy air was more prevalent among individuals who perceived moderate and high levels of annoyances than among individuals with a low level of annoyances [38]. Given that individuals with moderate and high levels of annoyances had increased IR for respiratory infections, our findings went in the same direction.

Our findings were in line with all of the previous studies [15,16,17,18,32]. We found an increase in the broad category of respiratory infections as reported in previous studies [15,16,17,18,32] and a non-significant decrease in viral infections in individuals with a high level of annoyances compared to those with a low level of annoyances [32].

This appears to be the first study to examine the association between perceived annoyances and bacterial respiratory infections, as well as respiratory infections leading to hospital admissions among individuals over 16 years of age. Furthermore, this study investigated the association between annoyances and respiratory infections in a population with asthma and COPD. For that reason, comparison with previous research is limited. The literature concerning indoor environmental exposures and the association with hospital admissions for respiratory infections mainly focuses on children and adolescents. However, one meta-analysis demonstrated that indoor and outdoor air pollutants were associated with significantly increased risk of pneumonia-specific hospital admissions or emergency room visits among individuals in all ages [52].

### 4.3. Interpretations

The perception of annoyances has been found to be moderated by certain demographic, social, personal, and situational factors [53,54]. Therefore, it is noteworthy that the full effect cannot be solely ascribed to the source itself. Our data revealed demographic and social differences across individuals who perceived low, moderate, and high levels of annoyance, while we were not able to investigate personal factors. However, identifying the effects of single factors on annoyances is challenging because they are not independent of each other [53].

In the present study, we found associations but we were unable to investigate the underlying pathways. The associations might be a result of annoyances, yet it is also possible that people with poorer health have an increased risk of being annoyed in their indoor environment at home, to report annoyances, or spend more time at home. Individuals might also attribute health symptoms to annoyances or other negative health aspects to the indoor environment at home, and, therefore, report annoyances.

On average, adults have 2–4 common colds per year [55]. In the present study, individuals had on average 11.3 and 9.1 respiratory infections that required treatment per 100 person years. This is fewer than the estimated yearly average, but was expected, as we lack information on common colds. However, as those infections included in our study were well-defined via registers, we were still able to make valid conclusions. Furthermore, the results were robust in the sensitivity analyses that applied a different outcome definition. Whether those who receive medical attention (i.e., included in the present study) also had the most common colds is, to our knowledge, unknown.

In the present study, diagnoses of viral respiratory infections and respiratory infections causing hospital admission were relatively rare. This might have influenced the lack of significant differences for those with high and moderate levels of annoyances compared to those with a low level of annoyances. Furthermore, there is symptom overlap between influenza and bacterial infections, which makes it difficult to clinically distinguish whether an influenza patient has a bacterial coinfection [28]. We hypothesize that the substantially higher IR for bacterial respiratory infections compared to that for viral infections is due to caution from doctors. Thus, we expect that the number of prescriptions is an overestimation of the actual number of bacterial respiratory infections. Increased awareness of antibiotic resistance has led to a range of interventions to reduce inappropriate prescriptions [56,57,58]. As a result, there were decreases in the use of beta-lactamase-susceptible penicillin and macrolide in general practice from 2004 to 2013 [59]. The number of viral infections, however, was most likely underestimated since these are self-limited illnesses [27] that resolve without medical treatment, meaning that most cases do not lead to hospital contact.

The analyses of individuals with asthma or COPD had non-significant IRRs; the IRRs were increased for a high level of annoyances and decreased for a moderate level of annoyances compared to a low level of annoyances, which might point towards no association. However, in addition to a gradual increase in exposure from low to high levels of annoyances, the levels were also characterised differently since the statistical analysis grouped individuals based on similarities in their indoor pattern of annoyance. Individuals with a high level of annoyances were especially annoyed by temperature and draught [38], which might have driven the implied effect. In contrast, individuals with a moderate level of annoyances were mainly characterised by annoyances from traffic noise, neighbour noise, and vibrations in buildings [38], and the association to respiratory infections did not seem to differ from that of individuals with few mixed annoyances (low level). Furthermore, it is noteworthy that there were a rather low number of participants with asthma and COPD and thus rather few respiratory infections. This means that we cannot conclude that there was no impact of perceived annoyances for these groups, as our study was statistically much weaker for these groups. This may also be true regarding the low number of diagnosed viral infections and hospital admissions among individuals without asthma or COPD.

The results differed between the sensitivity analyses that tested the effect of asthma or COPD diagnosis before and after study entry. Highly annoyed individuals diagnosed after baseline had a higher IRR than those diagnosed before study entry when compared to those with a low level of annoyances. This might be due to baseline differences between those diagnosed and not diagnosed, e.g., in perception, awareness on the indoor environment, time spend at home, and symptoms.

### 4.4. Strengths and Limitations

Previous studies have also shown increased incidence of respiratory infections at high annoyance, although these studies had several shortcomings that this study addressed in order to verify the validity of the findings. General differences include that none of the previous studies [15,16,17,18,32] used register-based outcomes, but instead used self-reported outcomes. Further, none of the previous studies [15,16,17,18,32] included exposures to multiple indoor contaminants. Although Niemann et al. (2006) used summary scores, it was solely for general traffic noise and neighbourhood noise, respectively [17], which limited the ability to reflect the complexity of indoor environments.

The population sizes differed in previous studies from 30 households [32], 104 teachers [15], 1142 individuals [18], 2674 individuals [16], and 8539 individuals [17], to 16,679 individuals in the present study, i.e., our study population was substantially larger than those of previous studies. Only one of the previous studies followed residents over time for six months [32], which enabled exposure to precede the outcome. Nevertheless, an effect may not necessarily show in short-term studies. Up to 19 years of risk time between exposure and outcome assessment allowed us to analyse recurrent events and include more cases of respiratory infections than previous studies.

Another key difference is that the statistical models of past studies were based on naive techniques (i.e., only using one observation for each individual) [60] and ignored recurrent events. We used a longitudinal technique that considered recurrent events and accounted for the fact that recurrent events for individuals are correlated [60] by applying a random effect and the number of previous events in the analysis.

We were able to link participants with individual-level information from Danish national registers, as all Danish residents are assigned a unique personal identification [61]. Therefore, we had access to information on outcomes and covariates. Diagnoses of pneumonia (J12–J18) in the Danish National Patient Registry have shown to have a positive predictive value of 92.9% (95% CI: 66.1, 99.8) by review of medical records [62]. The Danish National Patient Registry has also been found to be a promising tool for respiratory syncytial virus (RSV; J12.1, J20.5, J21.0, B97.4) research with 68% of patients having a proven RSV disease [63]. To our knowledge, no validation studies have reviewed respiratory infections by comparing against prescriptions from the Danish National Prescription Registry. However, pharmacies and hospitals receive a financial incentive for complete registration of all purchases and hospital contacts via a reimbursement system and are thus considered complete [39,40,64].

Our study had unique information on self-reported behaviours and perceptions, which provided detailed information on the exposures and enabled us to adjust the analyses, e.g., for smoking status. Furthermore, it had very few missing data for the variables used to create the exposures and for the confounders, which was likely a result of the interview-based study design.

Another strength is that the study population was based on a representative, randomly selected sample of individuals over 16 years of age, which allowed us to generalise the findings to all Danish individuals over 16 years of age. In addition, the participation response was high, with participation by almost three of four invited individuals; however, participants and non-participants may differ. Thus, we used weighted regression methods to account for non-participation and minimise that selection bias may have influenced the findings.

Last, we were able to stratify our analyses by asthma/COPD status. Hence, the study population became rather homogenous, so associations were less likely to be confounded by a substantial variation in perceived annoyances and risk between individuals with and without asthma or COPD. No previous study in this field has performed analyses based on the study population’s respiratory health status.

A major limitation of our study was that the level of perceived annoyances was collected at a single point in time, thus we cannot model changes in the level of perceived annoyances, for example, based on improvements to the indoor environment or changes to the outdoor environment, such as new neighbours or road construction. Even changes from season or time of day may influence the responses. We acknowledge that more than one collection of perceived annoyances per individual would have helped us understand whether annoyances were a static perception or not, thereby affecting the validity of our findings. Measuring the level of perceived annoyances was further limited, as the questions for perceived annoyances were not based on standardised specifications nor validated. Nevertheless, as annoyances are a subjective perception, the self-reported questions may not cause negative effects on the study. A recent study showed that perceptions of the indoor air and thermal comfort were correlated with the measured results from the monitors [65]. The advantages of surveys compared to measurements of a stationary monitor are that they are non-intrusive, cost-effective, and might provide better representation of the actual indoor environment since they might take account of a wide range of factors that are difficult to measure directly [32,66]. However, we acknowledge that we cannot exclude a bias from unmeasured or unrecognised biases.

Another limitation was that our outcomes only included information on events where the individual was diagnosed at a hospital or redeemed prescription drugs. Therefore, we lack data from medical practices and laboratory tests to better estimate the number of viral respiratory infections. Nevertheless, even with this information, we most likely still missed cases of viral infections since many individuals stay home when sick without contacting a doctor. This might have biased the effect of the perceived annoyances, yet the misclassification is non-differential.

### 4.5. Implications and Impact

We found that individuals who perceived moderate and high levels of annoyances in their home had more cases of respiratory infections than individuals with low levels of annoyance. Few other studies have investigated the association between perceived annoyances in the indoor environment and respiratory infections among adults [15,16,17,18,32]. To our knowledge, we are the first to study to report an association between exposure to multiple environmental annoyances and respiratory infections. Furthermore, this is the first report to study individuals with and without asthma or COPD separately and assess whether annoyances are associated with bacterial respiratory infections and hospital admissions caused by respiratory infections. Therefore, further studies are needed to confirm the findings in the present study.

Further studies of perceived annoyances in the home environment and the impact on health can be used to identify potential interventions or contribute to the development a perception-based approach to evaluate risk of disease [67]. Policymakers should support the discussion of annoyances between planners and residents in housing development, construction, and rehabilitation to reduce annoyance ratings [53,68]. Further, policy makers should support planners keeping themselves informed and incorporate health considerations into house planning [68].

## 5. Conclusions

In summary, perceived annoyances were associated with respiratory infections overall and bacterial respiratory infections in a cohort of individuals without asthma or COPD. In addition, associations were not found for viral respiratory infections and hospital admissions. Individuals with asthma or COPD and a high level of perceived annoyances had non-significantly increased for all four types of respiratory infections compared to those with a low level of annoyances. Based on the significantly increased IR for respiratory infections of all causes, these findings provide support for perceived annoyances as an important risk factor for respiratory infections. Few studies examining the association between perceived annoyances and respiratory infections exist, and the present study investigated new aspects, e.g., we separated the analyses based on asthma/COPD status and applied a stronger methodological design than previous studies. Thus, further studies are needed to confirm the findings.

## Figures and Tables

**Table 1:** Baseline characteristics of participants (total *n* = 16,679) and their dwellings by level of perceived annoyances in the indoor environment. Values are numbers and percentage in columns.

Baseline Characteristics		*n* (%)	Level of Annoyances, *n* (%)		
			Low (*n* = 14,820)	Moderate (*n* = 980)	High (*n* = 879)
Participants					
Sex	Male	8181 (49)	7307 (49.3)	503 (51.3)	371 (42.2)
	Female	8498 (51)	7513 (50.7)	477 (48.7)	508 (57.8)
Age group, years	<20	876 (5.3)	771 (5.2)	35 (3.6)	70 (8)
	20–24	1307 (7.8)	1053 (7.1)	104 (10.6)	150 (17.1)
	25–29	1366 (8.2)	1107 (7.5)	112 (11.4)	147 (16.7)
	30–34	1452 (8.7)	1241 (8.4)	92 (9.4)	119 (13.5)
	35–39	1485 (8.9)	1311 (8.9)	97 (9.9)	77 (8.8)
	40–44	1512 (9.1)	1351 (9.1)	106 (10.8)	55 (6.3)
	45–49	1554 (9.3)	1408 (9.5)	85 (8.7)	61 (6.9)
	50–54	1628 (9.8)	1492 (10.1)	86 (8.8)	50 (5.7)
	55–59	1425 (8.5)	1317 (8.9)	75 (7.7)	33 (3.8)
	60–64	1059 (6.3)	967 (6.5)	54 (5.5)	38 (4.3)
	65–69	866 (5.2)	798 (5.4)	42 (4.3)	26 (3)
	70–74	753 (4.5)	708 (4.8)	28 (2.9)	17 (1.9)
	75–79	636 (3.8)	583 (3.9)	34 (3.5)	19 (2.2)
	80–84	433 (2.6)	404 (2.7)	20 (2)	9 (1)
	≥85	327 (2.0)	309 (2.1)	10 (1)	8 (0.9)
Education ^a^	Elementary	6368 (38.2)	5689 (38.4)	321 (32.8)	358 (40.7)
	Short	6997 (42)	6185 (41.7)	452 (46.1)	360 (41)
	Medium/long	3304 (19.8)	2937 (19.8)	206 (21)	161 (18.3)
Smoking status	Smoker	6184 (37.1)	5382 (36.3)	377 (38.5)	425 (48.4)
	Non-smoker	10,470 (62.8)	9413 (63.5)	603 (61.5)	454 (51.7)
	Missing	25 (0.1)	25 (0.2)	0 (0)	0 (0)
Country of origin	Danish	16,349 (98)	14,532 (98.1)	959 (97.9)	858 (97.6)
	Non-Danish ^b^	330 (2)	288 (1.9)	21 (2.1)	21 (2.4)
Asthma or COPD diagnosis	Yes, *n* (%)	1153 (6.9)	1029 (6.9)	52 (5.3)	72 (8.2)
	No, *n* (%)	15,526 (93.1)	13,791 (93.1)	928 (94.7)	807 (91.8)
Household and Environment					
Type of dwelling	Detached house	8523 (51.1)	7867 (53.1)	419 (42.8)	237 (27)
	Semi-detached and terrace houses	2831 (17)	2504 (16.9)	157 (16)	170 (19.3)
	Apartments	3427 (20.5)	2713 (18.3)	344 (35)	370 (42.1)
	Other house type	1360 (8.2)	1272 (8.6)	30 (3.1)	58 (6.6)
	Farms	435 (2.6)	375 (2.5)	24 (2.5)	36 (4.1)
	Missing	103 (0.6)	89 (0.6)	6 (0.6)	8 (0.9)
Degree of urbanisation	<200 residents	2631 (15.8)	2431 (16.4)	91 (9.3)	109 (12.4)
	200–4900 residents	4228 (25.3)	3884 (26.2)	203 (20.7)	141 (16.04)
	5–49,000 residents	4984 (29.9)	4449 (30)	283 (28.9)	252 (28.7)
	≥50,000 residents	4195 (25.2)	3485 (23.5)	366 (37.4)	344 (39.1)
	Missing	641 (3.8)	571 (3.9)	37 (3.8)	33 (3.8)
Heating source	Direct electricity	1254 (7.5)	1150 (7.8)	49 (5)	55 (6.3)
	Central heating from liquid fuel	3581 (21.5)	3251 (21.9)	169 (17.2)	161 (18.3)
	Central heating from natural gas	2614 (15.7)	2373 (16)	152 (15.5)	89 (10.1)
	Other, gas furnace, solid fuel stove	455 (2.7)	408 (2.8)	22 (2.2)	25 (2.8)
	Undisclosed	8775 (52.6)	7638 (51.5)	588 (60)	549 (62.5)
Residential density, m^2^/resident	<40	2748 (16.5)	2501 (16.9)	164 (16.7)	83 (9.4)
	40–79	7691 (46.1)	6948 (46.9)	413 (42.1)	330 (37.5)
	≥80	5541 (33.2)	4751 (32.1)	364 (37.1)	426 (48.5)
	Missing m^2^	699 (4.2)	620 (4.2)	39 (4)	40 (4.6)
Number of residents in household	1	3349 (20.1)	2916 (19.7)	225 (23)	208 (23.7)
	2	6358 (38.1)	5644 (38.1)	405 (41.3)	309 (35.2)
	3	2829 (17)	2526 (17)	150 (15.3)	153 (17.4)
	4	2723 (16.3)	2442 (16.5)	136 (13.9)	145 (16.5)
	≥5	1420 (8.5)	1292 (8.7)	64 (6.5)	64 (7.3)
Years lived in the dwelling	<3	4841 (29)	4054 (27.4)	383 (39.1)	404 (46)
	3–7	3691 (22.1)	3245 (21.9)	222 (22.7)	224 (25.5)
	8–12	2035 (12.2)	1850 (12.5)	93 (9.5)	92 (10.5)
	13–20	2595 (15.6)	2397 (16.2)	107 (10.9)	91 (10.4)
	≥21	3517 (21.1)	3274 (22.1)	175 (17.9)	68 (7.7)
Interview Details					
Season of interview	Spring	4404 (26.4)	3951 (26.7)	286 (29.2)	167 (19)
	Summer	3166 (19)	2856 (19.3)	189 (19.3)	121 (13.8)
	Autumn	4309 (25.8)	3781 (25.5)	289 (29.5)	239 (27.2)
	Winter	4800 (28.8)	4232 (28.6)	216 (22)	352 (40.1)

Abbreviations: COPD, chronic obstructive pulmonary disease. ^a^ Missing *n* = 10; ^b^ Non-Danish consists of immigrants of western/non-western origin and descendants.

**Table 2:** Poisson regression of rates of recurrent respiratory infection among participants without asthma or COPD from the Danish Health and Morbidity Survey 2000.

Outcome	Level of Perceived Annoyances	*n*	Number of Events	PYs at Risk	IR Per 100 PY	IRR (95% CI) ^a^	Adjusted IRR (95% CI) ^a,b^
Respiratory infections of all causes ^c^							
	Low	13,787	13,949	124,602	11.19	1 (reference)	1 (reference)
	Moderate	928	875	7046	12.42	1.09 (0.98, 1.23)	1.15 (1.02, 1.31)
	High	806	584	4675	12.49	1.16 (1.02, 1.32)	1.16 (1.01, 1.34)
Bacterial respiratory infections ^d^							
	Low	13,787	13,074	124,645	10.49	1 (reference)	1 (reference)
	Moderate	928	826	7049	11.72	1.11 (0.99, 1.25)	1.17 (1.03, 1.33)
	High	806	559	4676	11.96	1.19 (1.04, 1.36)	1.18 (1.02, 1.37)
Viral respiratory infections ^e^							
	Low	13,787	1179	125,267	0.94	1 (reference)	1 (reference)
	Moderate	928	60	7089	0.85	0.84 (0.60, 1.17)	0.96 (0.66, 1.39)
	High	806	32	4704	0.68	0.65 (0.42, 0.99)	0.82 (0.50, 1.33)
Hospital admissions caused by respiratory infections ^f^							
	Low	13,787	855	125,593	0.68	1 (reference)	1 (reference)
	Moderate	928	40	7110	0.56	0.76 (0.50, 1.15)	0.93 (0.58, 1.50)
	High	806	20	4748	0.42	0.55 (0.32, 0.95)	0.74 (0.39, 1.40)

Abbreviations: COPD, chronic obstructive pulmonary disease; PY, person year; IR, incidence rate; IRR, incidence rate ratio (calculated using a Poisson regression model); CI, confidence interval. ^a^ Weighted for non-response and included a random effect. ^b^ Adjusted for sex, education, smoking status, number of residents, baseline years since moving in, dwelling type, degree of urbanisation, residential density, year of dwelling construction, season of interview, and calendar season. Further adjustments included: ^c^ Age (<33, 33–39, 40–44, 45–49, 50–54, 55–59, 60–64, 65–69, 70–74, 75–79, 80–84, ≥85), calendar year (2000–2002, 2003–2005, 2006–2008, 2009–2011, 2012–2014, 2015–2018), and number of previous respiratory infections (0, 1, 2, 3, 4, 5, 6, ≥7). ^d^ Age (<33, 33–39, 40–44, 45–49, 50–54, 55–59, 60–64, 65–69, 70–74, 75–79, 80–84, ≥85), calendar year (2000–2002, 2003–2005, 2006–2008, 2009–2011, 2012–2014, 2015–2018), and number of previous respiratory infections (0, 1, 2, 3, 4, 5, ≥6). ^e^ Age (<50, 50–59, 60–64, 65–69, 70–74, 75–79, 80–84, ≥85), calendar year (2000–2002, 2003–2005, 2006–2008, 2009–2011, 2012–2014, 2015–2018), and number of previous respiratory infections (0, 1, ≥2). ^f^ Age (<55, 55–64, 65–69, 70–74, 75–79, 80–84, ≥85), calendar year (2000–2002, 2003–2006, 2007–2010, 2011–2014, 2015–2018), and number of previous respiratory infections (0, 1, ≥2).

**Table 3:** Poisson regression of rates of recurrent respiratory infection among participants with asthma or COPD from the Danish Health and Morbidity Survey 2000.

Outcome	Level of Perceived Annoyances	*n*	Number of Events	PYs at Risk	IR Per 100 PY	IRR (95% CI) ^a^	Adjusted IRR (95% CI) ^a,b,c^
Respiratory infections of all causes ^d^							
	Low	2100	1476	16,297	9.06	1 (reference)	1 (reference)
	Moderate	105	63	782	8.06	0.80 (0.55, 1.18)	0.84 (0.59, 1.21)
	High	110	80	745	10.74	1.20 (0.85, 1.71)	1.20 (0.85, 1.69)
Bacterial respiratory infections ^e^							
	Low	2100	901	16,323	5.52	1 (reference)	1 (reference)
	Moderate	105	38	783	4.85	0.88 (0.64, 1.22)	0.80 (0.52, 1.23)
	High	110	48	746	6.43	1.17 (0.87, 1.56)	1.22 (0.82, 1.83)
Viral respiratory infections ^f^							
	Low	2100	634	16,343	3.88	1 (reference)	1 (reference)
	Moderate	105	25	784	3.19	0.70 (0.55, 1.40)	0.76 (0.38, 1.49)
	High	110	33	747	4.41	1.11 (0.59, 2.06)	1.28 (0.68, 2.42)
Hospital admissions caused by respiratory infections ^e^							
	Low	2100	547	16,367	3.34	1 (reference)	1 (reference)
	Moderate	105	22	785	2.80	0.70 (0.33, 1.45)	0.79 (0.39, 1.61)
	High	110	28	749	3.74	1.14 (0.59, 2.19)	1.31 (0.68, 2.53)

Abbreviations: COPD, chronic obstructive pulmonary disease; PY, person year; IR, incidence rate; IRR, incidence rate ratio (calculated using a Poisson regression model); CI, confidence interval. ^a^ Weighted for non-response and included a random effect. ^b^ The adjustment set did not include year of dwelling construction due to lack of converge of the model when included. ^c^ Adjusted for sex, education, smoking status, number of residents, baseline years since moving in, dwelling type, degree of urbanisation, residential density, season of interview, calendar season, calendar year, and baseline asthma/COPD. Further adjustments included: ^d^ Age (<38, 38–44, 45–49, 50–54, 55–59, 60–64, 65–69, 70–74, 75–79, 80–84, ≥85) and number of previous respiratory infections (0, 1, 2, ≥3). ^e^ Age (<38, 38–44, 45–49, 50–54, 55–59, 60–64, 65–69, 70–74, 75–79, 80–84, ≥85) and number of previous respiratory infections (0, 1, ≥2). ^f^ Age (<60, 60–64, 65–69, 70–74, 75–79, 80–84, ≥85) and number of previous respiratory infections (0, 1, ≥2).

## Data Availability

The data presented in this study are only available anonymised by Statistics Denmark, as Danish law imposes restrictions to protect privacy. Any data access requests should be addressed to Statistics Denmark.

## Supplementary Materials

The following supporting information can be downloaded at: https://www.mdpi.com/article/10.3390/ijerph20031911/s1, Figure S1: Simplified Directed Acyclic Graph (DAG) highlighting variables of importance in the analysis of the association between experienced annoyances in the indoor environment at home and the number of respiratory infections since 2000; Table S1: ICD-10 and ATC codes for respiratory infections; Table S2: Asthma and COPD definitions.
