# Supplementary material for: The Association between Perceived Annoyances in the Indoor Home Environment and Respiratory Infections: A Danish Cohort Study with up to 19 Years of Follow-Up
Source: Int J Environ Res Public Health. 2023 Jan 20;20(3):1911. doi: 10.3390/ijerph20031911 (PMC9915003; doi:10.3390/ijerph20031911)
Supplement: Supplementary file 1 [file ijerph-20-01911-s001.zip › Figure S1.pdf]

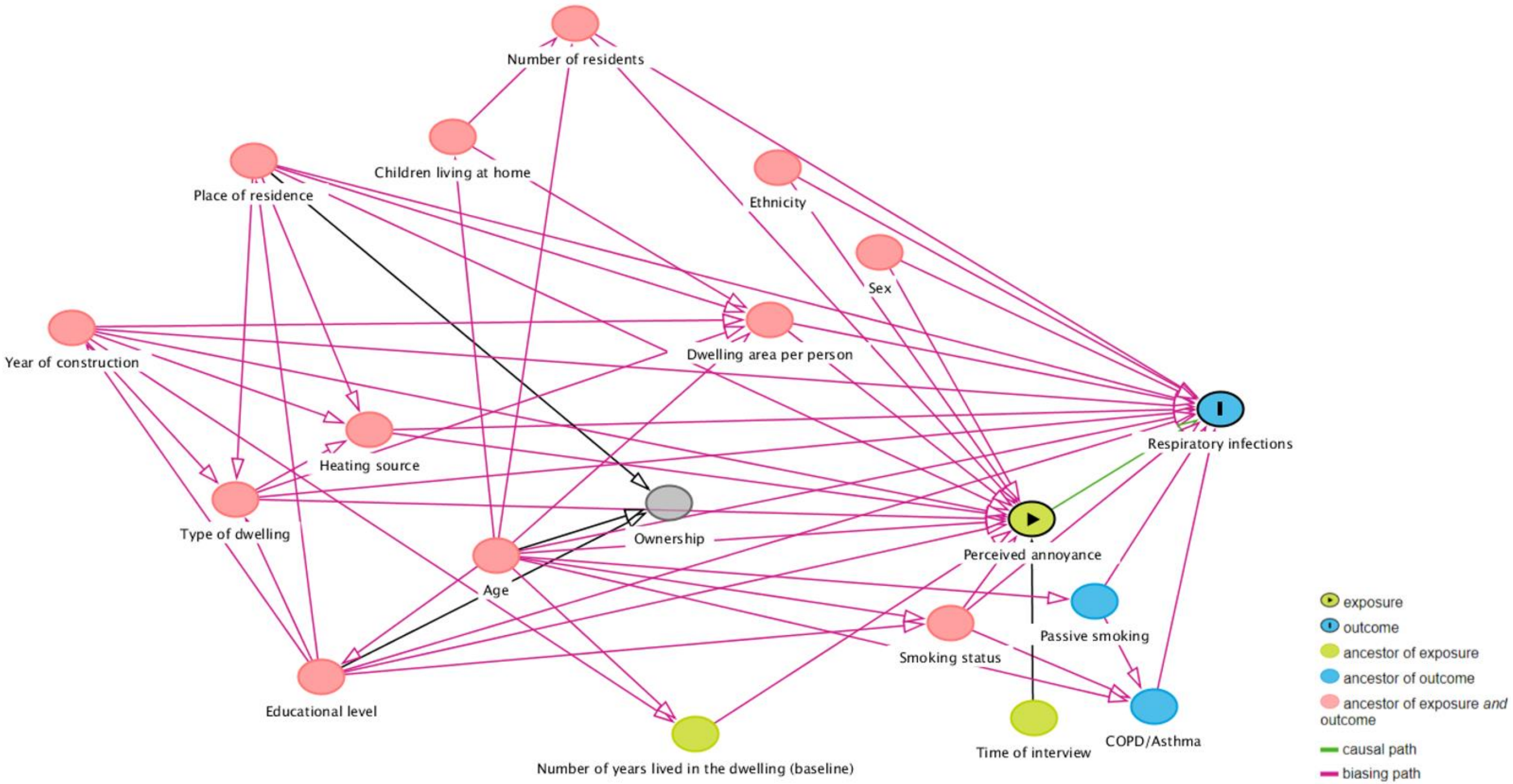


**Figure S1.** Simplified Directed Acyclic Graph (DAG) highlighting variables of importance in the analysis of the association between experienced annoyances in the indoor environment at home and the number of respiratory infections since 2000.
