# Supplementary material for: The Association between Perceived Annoyances in the Indoor Home Environment and Respiratory Infections: A Danish Cohort Study with up to 19 Years of Follow-Up
Source: Int J Environ Res Public Health. 2023 Jan 20;20(3):1911. doi: 10.3390/ijerph20031911 (PMC9915003; doi:10.3390/ijerph20031911)
Supplement: Supplementary file 1 [file ijerph-20-01911-s001.zip › Table S1.pdf]

Table S1. ICD-10 and ATC codes for respiratory infections

	Specification
**ICD-10 code** [a]	J00; J01; J02; J03; J04; J05; J06; J09; J10; J11; J12; J13; J14; J15; J16; J17; J18; J20; J21; J22: R05; R07.0
**ATC codes** [a]	R01[b, c, d]; R02[b]; R03[b, d]; R05[b, d]; R06[b, c]; R07[b]; J01CE02; J01FA01[e]; J01FA06[e]; J01FA09[e]; J01FA10[e]

Abbreviations: ICD-10, International Classification of Diseases and Related Health Problems 10th revision; ATC, Anatomical Therapeutic Chemical.

[a] All sublevels are included; [b] Only short-term uses are included (defined as one prescription within the last 12 months); [c] Except if individuals are diagnosed with allergic rhinitis (ICD-10: J30); [d] Except if individuals are diagnosed with asthma or COPD (see definition in Supplemental Table 2); [e] Included in the first sensitivity analyses.
