# Supplementary material for: The Association between Perceived Annoyances in the Indoor Home Environment and Respiratory Infections: A Danish Cohort Study with up to 19 Years of Follow-Up
Source: Int J Environ Res Public Health. 2023 Jan 20;20(3):1911. doi: 10.3390/ijerph20031911 (PMC9915003; doi:10.3390/ijerph20031911)
Supplement: Supplementary file 1 [file ijerph-20-01911-s001.zip › Table S2.pdf]

Table S2. Asthma and COPD definitions

	Data source	Specification
**COPD [a]**	**ICD-8**	490-492.
	**ICD-10**	J44 as primary diagnosis.
		J96 as primary diagnosis in combination with J44 as secondary diagnosis.
		J13-J18 as primary diagnosis in combination with J44 or J96 as secondary diagnosis.
	**ATC codes**	All prescriptions with indication code 379 and 464
		R03A.
		R03B.
		Redemption of two of the above prescriptions within one year.
**Asthma**	**ICD-8**	493.
	**ICD-10**	J45.
		J46.
	**ATC codes**	All prescriptions with indication code 202, 203 and 822.
		R03DC03.
		Redemption of two of the above prescriptions within one year.

Abbreviations: COPD, chronic obstructive pulmonary disease; ICD-8, International Classification of Diseases 8th revision; ICD-10, International Classification of Diseases and Related Health Problems 10th revision; ATC, Anatomical Therapeutic Chemical.

[a] Only individuals aged 30 years or above are included
